# Active Electric Imaging: Body-Object Interplay and Object's “Electric Texture”

**DOI:** 10.1371/journal.pone.0022793

**Published:** 2011-08-18

**Authors:** Ángel A. Caputi, Pedro A. Aguilera, Ana Carolina Pereira

**Affiliations:** Department of Integrative and Computational Neurosciences, Instituto de Investigaciones Biológicas Clemente Estable, Montevideo, Uruguay; University of California Davis, United States of America

## Abstract

This article deals with the role of fish's body and object's geometry on determining the image spatial shape in pulse Gymnotiforms. This problem was explored by measuring local electric fields along a line on the skin in the presence and absence of objects. We depicted object's electric images at different regions of the electrosensory mosaic, paying particular attention to the perioral region where a fovea has been described. When sensory surface curvature increases relative to the object's curvature, the image details depending on object's shape are blurred and finally disappear. The remaining effect of the object on the stimulus profile depends on the strength of its global polarization. This depends on the length of the object's axis aligned with the field, in turn depending on fish body geometry. Thus, fish's body and self-generated electric field geometries are embodied in this “global effect” of the object. The presence of edges or local changes in impedance at the nearest surface of closely located objects adds peaks to the image profiles (“local effect” or “object's electric texture”). It is concluded that two cues for object recognition may be used by active electroreceptive animals: global effects (informing on object's dimension along the field lines, conductance, and position) and local effects (informing on object's surface). Since the field has fish's centered coordinates, and electrosensory fovea is used for exploration of surfaces, fish fine movements are essential to perform electric perception. We conclude that fish may explore adjacent objects combining active movements and electrogenesis to represent them using electrosensory information.

## Introduction

Electroreception is a sensory modality widely distributed in aquatic animals. A large proportion of fish species has electroreceptor mosaics that sense electric fields across their skins. These transcutaneous patterns constitute electric images of the nearby environment [1]–[3].

Fish's electroreception has two modes, passive and active. In the passive mode, electric images are the transcutaneous fields caused by external sources [4]. In the active mode, the signals are carried by a self-generated electric field [5]. Nearby objects differing in conductivity from water are polarized by such self-generated field, behaving as virtual electric sources. As the objects illuminated by the sun light project their images on the retina, objects polarized by self-generated electric fields project their electric images on the electroreceptive mosaic at the skin.

General rules predicting the formation of active electroreceptive images were first formalized by Lissmann and Machin [6] and further extended by computer modeling [7]–[19]. Some of these rules were verified experimentally [20]–[27]. Most studies have been focused on the fish's side. However, after the discovery of electrosensory foveae [28], the exploration of object's images on the head became essential for understanding active electric sense. These foveae are sensory mosaic specializations (high receptor density, variety and central representation) observed at the perioral region of most electric fish [28]. Strikingly, *Gnathonemus petersii*, have two foveae. One of them is mounted on a mobile chin appendix (also called “schnauzenorgan” or “barbillon”) that gifts these animals with the additional ability to explore the objects by moving this appendix like a finger [29]–[34].

Several studies stress the importance of the fish's body as a polarizable object, inextricably present in any active electrosensory scene [10], [11], [14], [25], [35]. Sicardi et al. [11] stressed the importance of curvatures and edges for determining electric images. Even in fish lacking schnauzenorgan, modeling electric images at the head is very difficult because of the geometric detail of this region where the surface sinuosity caused by the mouth, olfactory papillae, eyes, opercular apertures and lateral line channel orifices are prominent. These geometric accidents are associated to variations in skin resistance which may also contribute to altering the local field [36]. These features cannot be accounted by modeling and this induced us to study the roles of the fish's body by measuring the electric images of objects.

This study is focused on *Gymnotus omarorum*, a species in which we had first described an electrosensory fovea [28]. The field vector in the fovea's neighborhood is characterized by a site-to-site waveform coherence and a fix direction during the time course of the electric organ discharge (EOD) [24], [25]. At the fovea, these characteristics of the active electrosensory carrier allow the fish to detect and discriminate small changes in the time course of the local waveform caused by the presence of complex impedance objects (i.e. showing capacitive and resistive components [26]). On the rest of the body, the distributed nature of the electric organ associated to the differences in time course of the regional EOD causes wobbling, site-specific, loops in the transcutaneous field [24].

This article deals with the role of fish's body and object's geometry on determining the image spatial shape. It pays special attention to the following questions concerning the image formation at the foveal region: a) does body geometry affect the electric image profile? b) how are object's geometric features represented in the spatial profiles of the electric images?

Measurements of the electric images of single objects differing in shape and size and placed at various positions relative to the fish's body indicate that: a) at the fovea spatial energy profiles in the presence of an object are importantly determined by body curvature; b) these spatial profiles are also dependent on the curvature of the object and show peaks when object's edges face the skin; c) image maximal amplitude depends on the object length along the field lines and on the distance between the facing surfaces of the object and the skin. We concluded that objects are represented in the image through two main effects: one global and other local. While the global effect carries information about large objects sensed distant from the fish, the local effects provide fine information about the presence of small objects or about the surface of large objects located very close to the skin.

## Results

### The effects of body shape on the spatial profiles of *rms*LEOD

To study electric images we sampled the stimulus of the receptive mosaic (local self-generated field, LEOD) along a line defined by the intersection of a horizontal plane with the fish body in the presence and absence of objects. We used two methods: a) measuring the orthogonal field components in the water adjacent to the fish skin (i.e. drop of voltage along longitudinal, transverse and vertical directions, 3D-LEOD), and b) measuring the transcutaneous drop of voltage (T-LEOD).

Both methods were complementary. The 3D-LEOD was useful in all regions of the skin but precluded the placement of the object at a distance from the skin closer than the electrode dimensions; the T-LEOD was only useful at the fovea, because, only at this region can a reference electrode be placed equidistant from the skin surface and relatively far away from the electrogenic sources. The small size of the electrode (150 micrometers in diameter) allowed exploring closer distances.

**Figure 1:**
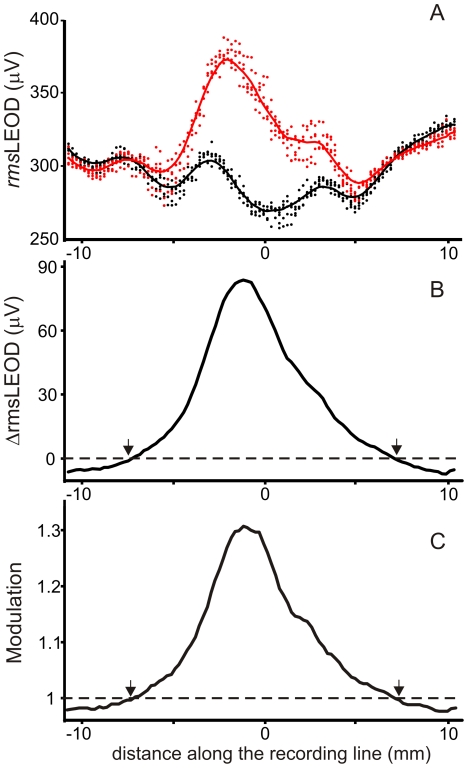
The image profiles at the fovea do not reflect the local variations in the stimulus patterns. Images of a sphere (16 millimeters placed 1 millimeter away from the skin) evaluated following the T-LEOD procedure A) *rms*LEOD profiles (dots, raw data; line, median moving averaged data) evaluated in the absence (black) and presence of the object (red). B) LEOD increment by the presence of the object C) LEOD modulation. Arrows indicate the transition between the top and the trough of the Mexican hat profile.

We represented the image of resistive objects as the change in the spatial patterns of the LEOD's root mean square value (*rms*). We chose arbitrarily a 10 ms window to evaluate this parameter, referred to as *rms*LEOD. Stimulus patterns are represented as *rms*LEOD plotted as a function of distance along the explored line. In the raw data these patterns were not smooth. Comparing run to run there were two types of variability: a) random (Fig. 1. A, raw data: dots), and b) repetitive spatial undulations. Random variability was eliminated by fitting the raw data with a smooth line calculated as a three or five point moving average of the median *rms*LEOD values obtained at each recording point (Fig. 1A, smoothed median value: continuous lines). We attributed these variations to the fact that recordings were obtained at different phases of respiratory cycle (see methods). Repetitive spatial undulations were present in all runs of the same trial. These undulations were characteristic of the fish and recording site. They remained in *rms*LEOD profiles evaluated in the absence (Fig. 1A, black line) and presence of the object (Fig. 1A, red line). This suggests that local accidents, (the mouth, the folding of the olfactory papillae, the openings of the lateral line canals, the eyes) may cause local variations of current density.

We evaluated the electric image as the increment or the modulation (difference or quotient between the smoothed median profiles of the *rms*LEOD). We plotted these profiles as a function of the distance along the skin (Fig. 1B and C, black lines). Interestingly, when the increment or the modulation profiles were calculated, the undulations were eliminated rendering a bell-shaped image. This indicates that the local undulations present in the raw stimulus patterns (in the presence or absence of an object) were minimized when calculating the electric image. For objects which orthogonal projections covered more than 10% of the transverse surface of the fish and were placed at the mid line, this bell-shaped image covers the foveal region entirely. The troughs of the Mexican hat effect were at the sides of the fish (N = 12, see the starting of the trough on the edge of the traces in Fig. 1, arrow).

**Figure 2:**
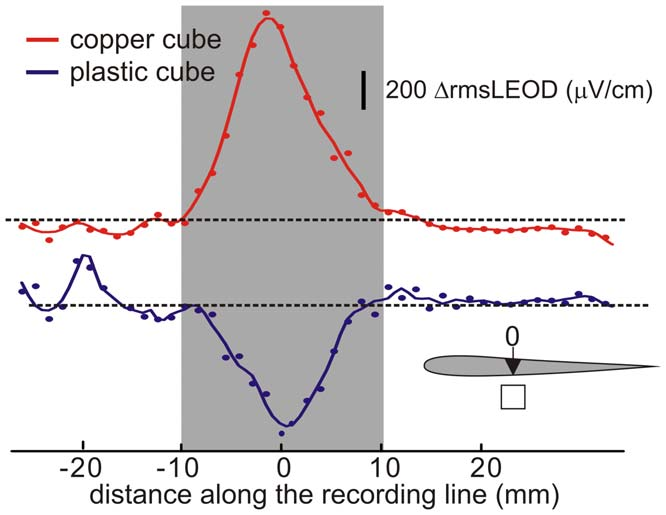
Electric images of a cube on the side of the fish. Images of a cube (20 millimeters side placed 4 millimeters away from the skin) evaluated following the 3D-LEOD procedure. Images consisted of a Mexican hat profiles. Top: A copper cube causes a center-increase surround-decrease of the *rms*LEOD pattern. Bottom: A plastic cube causes the opposite effect. Shadow area indicates the orthogonal projection of the cube. Dashed lines correspond to nulls Δ*rms*LEODs.

At the trunk, images were typical Mexican hat profiles. For example, a conductive cube (20 millimeters side, Fig. 2A) facing the skin 5 millimeters away shows a larger and inverted image than a non-conductive cube (Fig. 2B) otherwise identical in shape and positioning (image inversion when substituting metal by plastic objects of the same shape and position was observed in 12 experiments at the fovea and 12 at the trunk, Fisher exact test p<0.001).

The head and body regions are characterized by very different geometries. The shape of the head is a half ovoid while the fish body resembles a flattened cone. Thus, we studied separately object images on the head and on the side of the fish to evaluate whether the body shape has influence on the *rms*LEOD spatial profiles.

**Figure 3:**
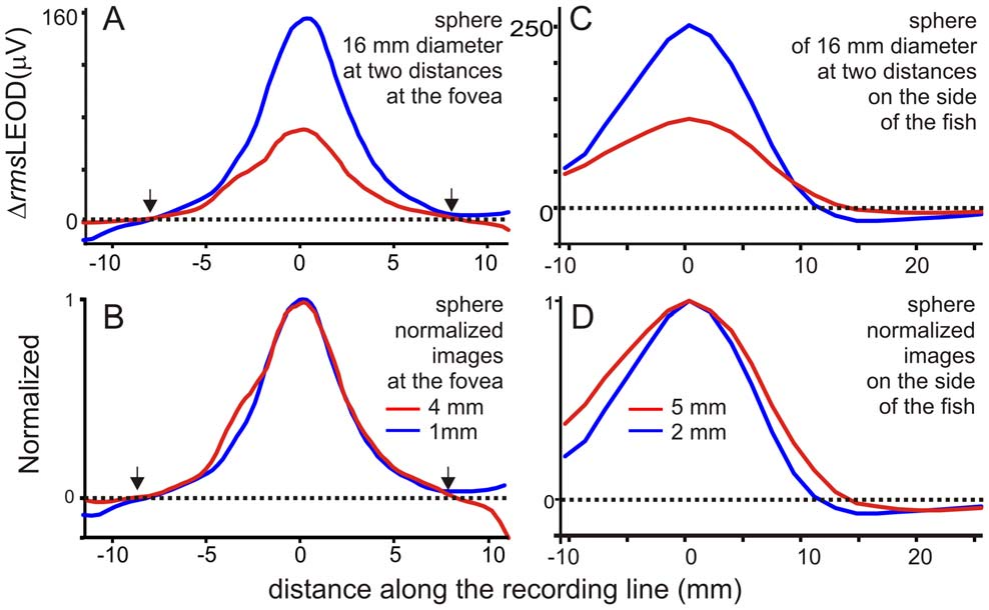
The effect of the fish body on the electric image. At the fovea, images are similar. A) Image of the same sphere at two distances (16 millimeters diameter, 1 and 4 millimeters away from the skin) showing a reduction in amplitude. B) Normalized profiles showing that the shapes of the profiles are identical. Arrows indicate the transition between the top and the trough of the Mexican hat profile. On the side of the fish, images increase in width when the object is moved away. C) The same sphere placed at two distances (16 millimeters diameter, 2.5 and 5 millimeters away from the skin) showing a reduction in amplitude. D) Normalized profiles showing that the image increased in width when the object was moved away.

On the head, images of spheres centered at the midline in front of the fovea showed similar *rms*LEOD normalized profiles. Moreover, when the object was moved away images diminished in amplitude (Fig. 3A) but normalized spatial profiles remained very similar (compare red and blue lines in Fig. 3B; in six paired experiments, differences in the relative slope were non-significantly different from zero, sign-test, p = 0.68). These similar bell-shaped profiles contrast with the distance- dependent Mexican hat profiles observed on the side of the fish's body (Fig. 3D). On the side, the width of the image increased with distance causing a reduction on the relative slope of the profile (sign-test, N = 6, p<0.05) as described previously [10], [11], [35] (Fig. 3C and D).

**Figure 4:**
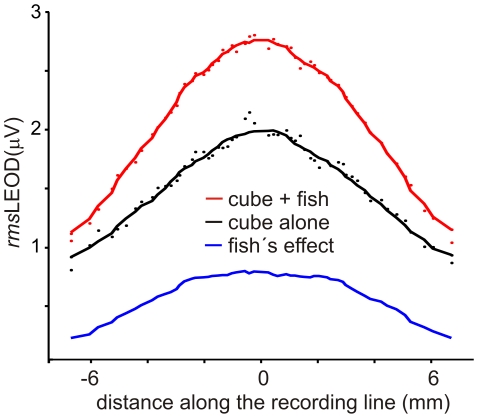
Effects of fish's body at the fovea. A) Field profiles recorded along a trajectory following the surface of the jaw of a fish's cadaver when a sinusoidal field was applied between the rostral and caudal walls of the tank. Red symbols represent the local field measured in the presence of a copper cube facing the fish's cadaver. Black symbols represent local field measured in the presence of the same object but in the absence of the fish's body. Blue line represents the effect of the fish's body calculated as the difference between the two fitted curves.

We hypothesized that these differences between the images of the same object at the rostrum and along a parasagittal plane on the fish's side are due to the body geometry. We postulate that for objects of similar or smaller curvature, body geometry dominates the profile yielding similar images. To show this geometrical effect we compared the image of a copper cube on the jaw of a fish's cadaver (Fig. 4 red dots and line) with the field profile at the same points in the absence of the fish's body (Fig. 4, black dots and line) when an external uniform field was applied between the rostral and caudal walls of the tank. The difference between both profiles was maximal at the center (40% of the maximum effect of the cube alone, Fig. 4, blue line) and minimal on the sides (less than 5%, Fig. 4).

### Global and local object's features are encoded in the *rms* profile at the fovea

Taking into account the sensory relevance of the foveal region, we investigated how three geometrical features of pure resistive objects are represented in the *rms*-LEOD profile: size, position and irregularities on the object surface facing the skin. We addressed three specific questions: a) how is object size represented? , b) how is object's position represented?, and c) is there something as an object's “electrical texture”?

With regard to the first question, we found that changes in object's size cause similar bell-shaped images with large differences in amplitude, but object's size is not faithfully encoded by amplitude.

**Figure 5:**
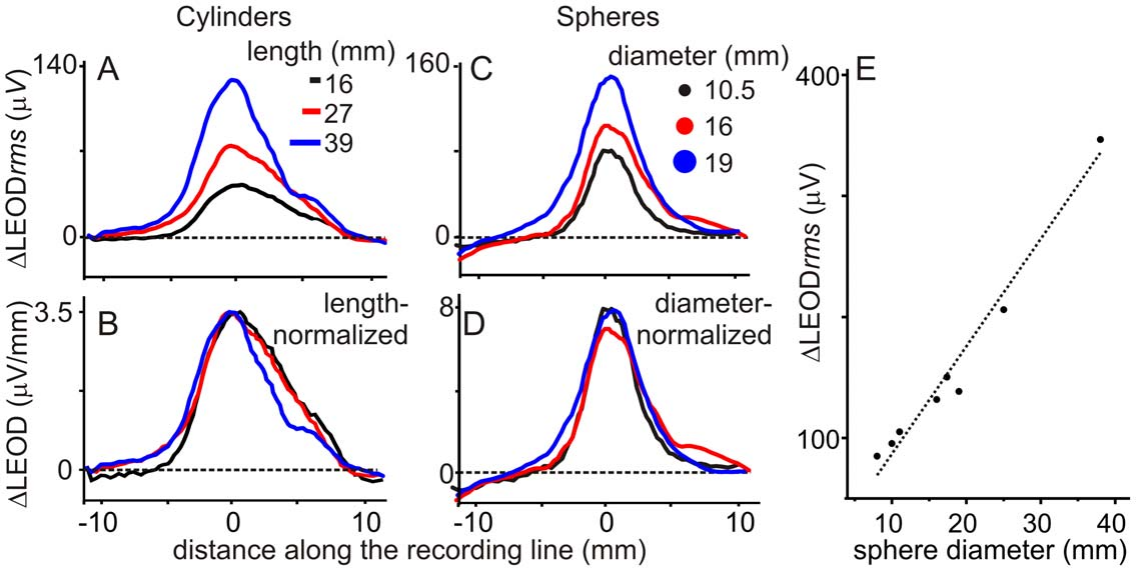
Amplitude of the global effect increases with the length of the field line covered by the object footprint. A and B) Images of cylinders of 10 millimeters diameter base and three different lengths with their main axis oriented along the field line. As the normalized profiles are the same the amplitude of the image grows linearly with the length of the cylinder. C and D) Image of three steel spheres showing that image amplitude increases linearly with the diameter but the normalized profile is the same. E) Image amplitude vs. diameter showing data from 8 spheres. r^2^ = 0.96 N = 8.

Image amplitudes of copper cylinders with equal bases facing the skin (10 millimeters diameter) increased linearly with cylinder length (Fig. 5A, similar data were obtained from 4 fish) and showed very similar length-normalized profiles (Fig. 5 B). Linear relationship and similarity of profiles were also found for spheres of diameters between 8 and 36 millimeters (Fig. 5C to E, similar data were obtained from 4 fish).

Nevertheless, orientation along the field lines may cause ambiguity. Theoretical analysis indicates that for the same distance between the surface of the object and the skin, image amplitude is a function of the polarization of the object and this, in turn, grows with the maximum difference in the basal field across the footprint of the object in its absence [35].

**Figure 6:**
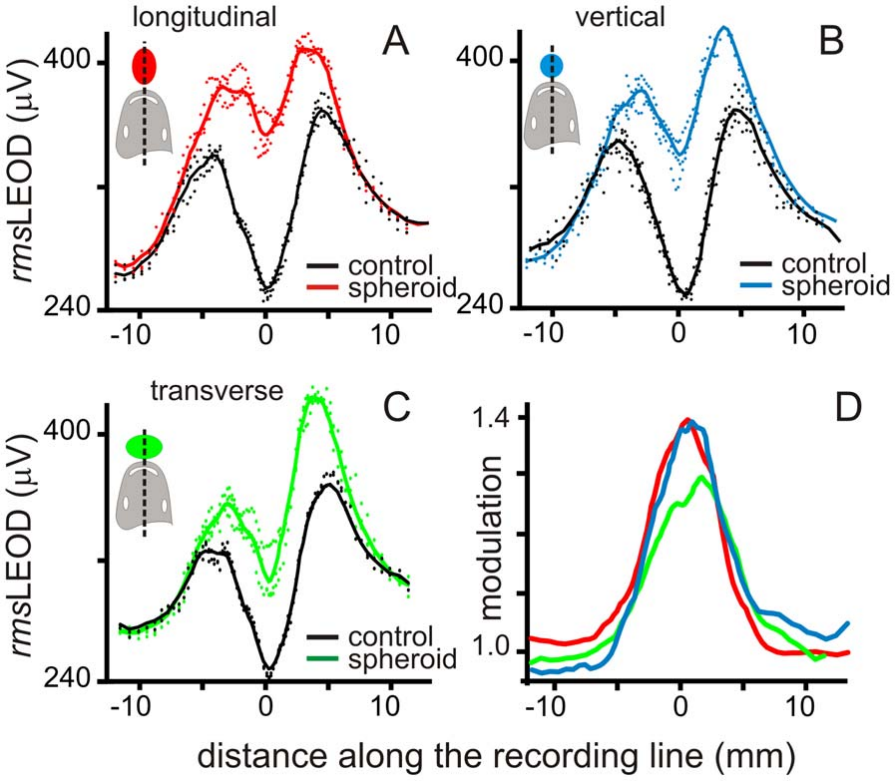
Importance of the alignment of the object with the field lines. Images of a prolate-spheroid-shaped copper object placed at the same distance but differently oriented with respect to the field. A to C: raw data (dots) and fitted stimulus profiles (lines). Object main axis orientation: A) longitudinal (red), B) vertical (blue), C) transverse (green). D) Modulation profiles show that in the transverse orientation (shorter dimension aligned with the field) the image of the smallest amplitude.

To explore whether the number of isopotential lines intersected by the object does matter for determining image amplitude we compared the images of a same prolate spheroid copper object (13.5×20 millimeters) with the long axis oriented in different directions in 3 fish. We predicted that the image of the same spheroid object should be larger when the longer axis is aligned with the field (affecting the largest number of isopotential lines). We verified this hypothesis by placing the spheroid with its surface at 1 millimeter away from the skin with its major axis orthogonally oriented (longitudinal, Fig. 6A red; vertical, Fig. 6B blue; or transverse, Fig. 6C green; referred to the main fish axis). Images were the largest when the basal field had the largest projection on the main axis of the object (longitudinal and vertical positions). To explain this result, one must take into account two physical features (see methods): i) the basal field is oriented upwards on the sagittal plane, thus vertical and longitudinally oriented objects receive the best projections of the strongest component of the field, and ii) the direction of this component tends to align with the major axis of a conductive object [35]. On the contrary when the object was transverse the projection of the largest component of the basal field was on the shortest dimension of the object and consequently the image was smaller (Fig. 6 D). Note that even though the orthogonal projection of the spheroid placed in the transverse position was larger along the recording line, the width of the image was the same as in the other positions, confirming the observation shown in Fig. 5. We conclude that object's size influences the amplitude of the image in two ways. First because a larger size would result in a larger change of local impedance; and second, elongated objects have an optimal orientation along the field lines.

**Figure 7:**
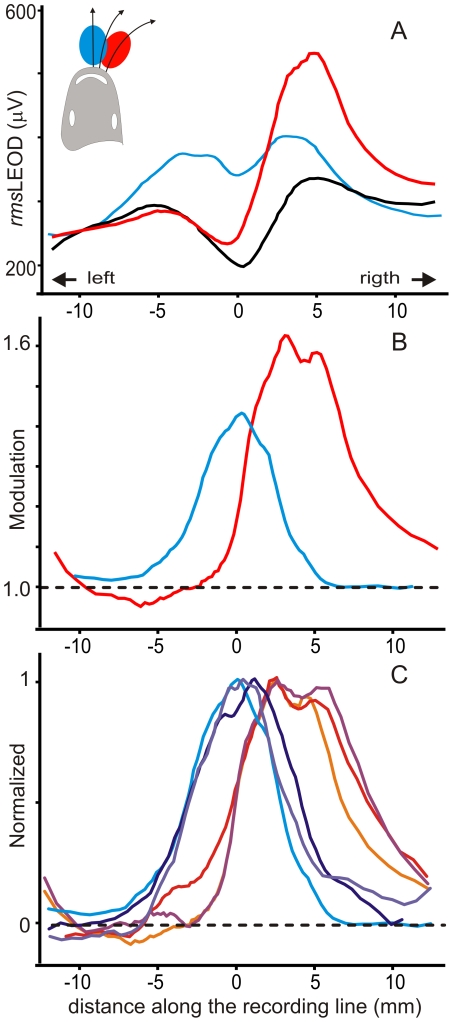
Image profiles depend on objects position. A) *rms*LEOD stimulus patterns in basal (black), object centered (blue) and object lateralized (red) are compared. The main axis of the object was aligned with the largest field component in each case. Note that when the object was on the side facing a peak of the basal profile the change was greater at the region facing the object and was reduced below the control line on the other side. B) Modulation profiles showed a larger peak and a contra-lateral through when the object was on the side. C) Superimposed normalized profiles obtained from six experiments in which the spheroid was differently oriented but with the closest point facing the same point at the skin (three placed at the middle cold color traces, and three on the side, warm color traces).

To address the second question, we compared the image of the spheroid with their main axis oriented along the midline with the image of the same object placed at the same distance from the skin but on the side of the head where the profile of the LEOD in the absence of the object showed a relative maximum (Fig. 7A, black trace).

In 3 animals the image of the copper prolate spheroid on the side of the head was clearly different in shape and amplitude from images obtained with the object centered at the midline. It showed a Mexican hat effect on the contra-lateral side and a broader profile (Fig. 7B). The modulation was the largest when the main axis of the object was oriented along the field facing a region where the basal LEOD was maximal (red lines Fig. 7A and B). In addition, when we compared the normalized images of the same ovoid placed facing either the midline or the side of the head but differently oriented (Fig. 7C; cold colors: middle, warm colors: side) similar but shifted profiles were obtained. This shift in the slope of the profile with object position suggests that the electrosensory system would receive the most information on object shape by image shifting flanks over the fovea using small head movements.

Finally, to address if object's surface features are represented at the fovea we explored the images of small objects and objects with differences in their surface.

**Figure 8:**
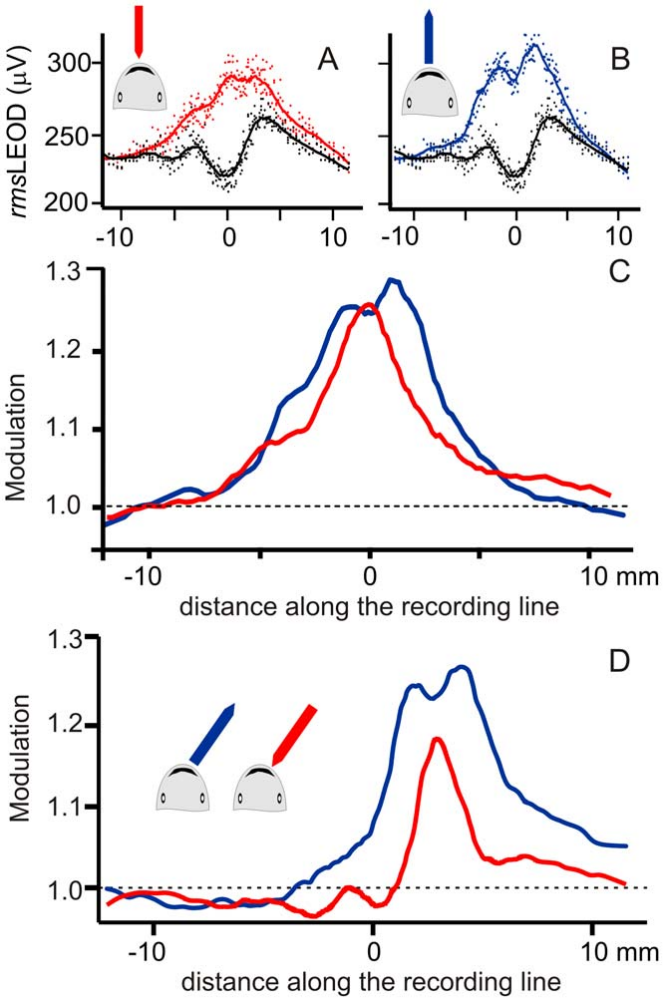
Edge effects on the image profiles. A and B) Raw data and fitting curve showing the raw stimulus profiles in the absence (black) and presence of a “nail shaped” object (3 millimeters diameter except for the conical end, 25 millimeters total length) oriented with the tip (red) or the back (blue) towards the fovea. C) Modulation profiles show a sharp peak in the tip facing condition and two peaks on top of the global effect profile corresponding to the limits of the circular base of the object. D) Similar effects when the objects were on the side of the head. Note the Mexican hat effect on the contra-lateral side.

**Figure 9:**
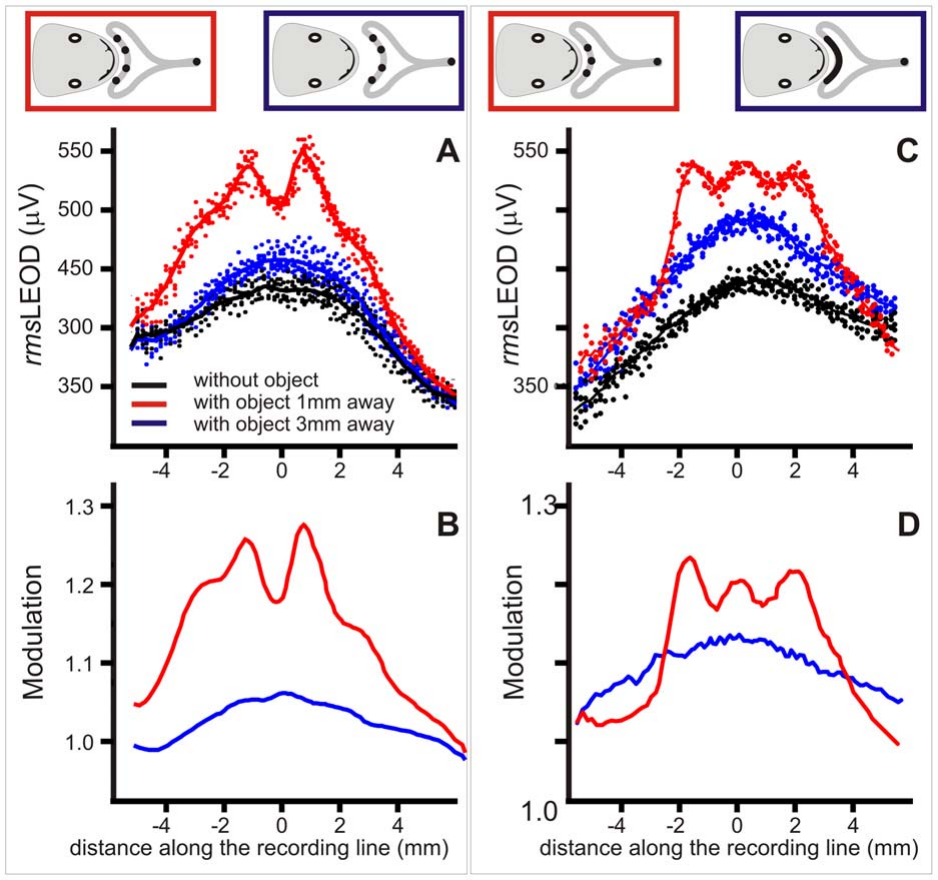
Surface electric features. We designed a special object to explore the local effects. This object consisted of a copper wire (1.5 millimeters diameter) shape covered with enamel except on the tips that were attached together at about 30 millimeters from the fish along the axis. Insets in left and right panels illustrate the experimental design. Plots A and B (corresponding to the red and blue outlined insets in this panel) illustrate the presence of the local effect and how it disappears at very short distances. In A the raw rmsLEOD data (dots) and the fitted curves (lines) are shown. B compares the modulation near (red) and far (blue) from the fish's body. The two large peaks at the center of the red lines and the small humps on the side were in beam with the points of the wire without varnish. Plots C and D (corresponding to the red and blue outlined insets in this panel) illustrate the different images of the same object having an “electrically textured” and an “electrically smooth” surface C) raw rmsLEOD data (dots) and the fitted curves (lines). D) Modulation. Three peaks at the center of the red lines were in beam with the points of the wire without varnish. These disappear when the varnish was removed all along the wire surface facing the skin.

Well defined profiles were obtained at the head region when small objects were placed very close to the skin. LEOD profiles show peaks corresponding to object's edges (N = 3, Fig. 8) or changes in conductance of their surface (N = 2, Fig. 9).

In figure 8, we compared the images generated by the presence of a nail-shaped steel object (3 millimeters diameter, 25 millimeters long) when either the sharpened or the blunt end faced the skin. When the flat base faced the skin the image was in average 2.1 times broader than when the edge faced the skin (N = 3, Fig. 8 A to C). In addition, due to the small diameter of the nail, experiments showed that the lateral edges of the head of the nail caused two relative maxima in the image.

When the object was lateralized, these edge effects were more marked and a small trough was more evident about the midline suggesting that the Mexican hat effect was now observed because of the lateralization of the object (Fig. 8D).

In figure 9, we pinpointed the differences between local and global effects of the objects described above by comparing objects with alternating conductive and non- conductive spots placed at two different distances from the skin (objects with textured surface). These effects disappeared when the object was moved away.

The example that best illustrates this point consisted of an experiment in which the exploring object consisted of wires adapted to the curvature of the fish's chin (schematized at the insets of Fig. 9). These wires (1.5 millimeters diameter) were insulated except at the tips that were attached together at 30 millimeters away from the fish.

In one experiment four small scratches of about 0.5–1 millimeters were made with a scalpel blade. We compared the stimulus patterns (*rms*LEOD, red object close, blue object far, black no object Fig. 9A).and the electric images (modulations, Fig. 9B) obtained when the objects were 1 millimeters and 3 millimeters apart. In the closest position, the image showed two clear peaks coincident with the central pair of scratches of the varnish and two small humps on the side. These features disappeared when the objects were moved 3 millimeters away from the skin surface (Fig. 9A and B).

In another experiment, we compared the stimulus patterns (*rms*LEOD, red three spots, blue bare wire, black no object Fig. 9C) and the electric images (modulations, Fig. 9D) obtained with a wire having three small scratches with those obtained with the same wire at the same position after entirely removing the varnish on the side facing the skin. The wire having three conductive spots projected a tri-modal image while the same bare wire projected a smooth and broader image with a smaller peak modulation (Fig. 9C and D).

## Discussion

### Body-object interaction

The cornerstone of electrolocation is that images result from the polarization of objects immersed in an electric carrier. When an object is placed in a uniform field, image amplitude increases as a function of the product between its volume and the departure in conductance from water [6], [9], [11], [35], [37]. Every object in a field is polarized according to its geometrical and impedance properties, introducing new information in the scene. This information takes the form of a secondary field (“object's perturbing field” [6]) that modifies the stimulus pattern on the skin (“the electric image of the object”). Thus, an object can be considered as equivalent to an electric source that adds a new field to the pre-existing one, this source is characterized by its “stamp” [35], [36]. In information theory words, the amount of the information that the object “sends” to the fish is contained in such object's stamp and is transmitted (obviously with decrement) along the distance between the object and the skin.

When a conductive element is placed in such a way that its footprint crosses more than one potential line in the basal field, it causes a short circuit. The more isopotential lines are crossed, the larger alteration of the field of potential will occur and the larger will be the stamp. Then, any object, in particular the fish's body, oriented following the field lines (i.e. perpendicular to the isopotential lines) will have the largest stamp.

To understand the effect of the fish's body, let's begin by considering the stamp of an object as a single dipole and the fish's as an elongated body of conductance much higher than water. The fish body is tail-tapered. Consequently, its conductance is the largest at the head. In addition, due to the alignment of the body and dipole axes, currents are funneled in the best way. Hence, when the head of the fish faces the object the reciprocal polarization of the fish's body and the object is maximal.

For objects having an orthogonal projection having a diameter larger than 30% of the fish head cross-section one, and placed facing the fovea on the midline, the image decays with distance but its bell-shaped profile is rather independent of the object's shape (Fig. 5). This is because current density is mainly determined by the curvature of the head. The transition between the “top center” and the “surrounding trough” is shifted caudally, beyond the body surface “visible” from the object. Caudal to the head the longitudinal resistance increases and the lateral resistance decreases relatively to longitudinal one. These and the presence of a low path at the gills cause a shift of the through caudal to the head region. Experiments in which the object was placed on one side of the head (Fig. 7 and 8) showed the through on the contra-lateral side supporting this view.

On the side of the fish the electric image has a Mexican hat profile similar to that described for Mormyrids [27]. When the object is close to the skin, the field is reoriented in such a way that the “top” region (i.e. the increase in the case of a conductive object or decrease in the case of a non conductive object) becomes nearly coincident with the orthogonal projection. When the object is moved away the images become blurred and the correlation between the image flanks and the limits of the objects deteriorates.

### Global effects

Real objects are often geometrically complex and are illuminated by non-uniform fields. Then, object's stamps are complex sources in spatial dimensions. One way of representing these complex sources in a three dimensional environment is using multipoles [38]. Then, object's stamp may be discomposed into a set of dipoles that generate the same field when substituting the object.

Large dipoles representing globally the object depend on departure of object conductance from water and the number of isopotential lines of the polarizing field covered by the object's footprint. This component of the image is referred to as “global effect” of the object. Large objects oriented along the field lines have large global stamps and are sensed from a longer distance from the skin. The contribution of these large dipoles to the image increases when they are moved close to the fovea but the shape of their images is very similar. The nearly invariant bell-shaped profile is mainly determined by the shape of the jaw.

Nevertheless, small movements of the head orienting differently the local electric field must cause dramatic changes in the position of the flanks of object's images (see Fig. 7). Image flanks generated when the object moves on the side of the head may enter and leave the fovea causing strong stimuli. In consequence, global effects are best suited to explore the limits of an object or to evaluate its size and global shape by re-orienting the head. Contrast side-to-side enhanced by the common mode rejection mechanism implemented by the contra-lateral inhibitory projections occurring at the electrosensory lobe [39] may contribute to detect the objects' contour.

Global effects are also suitable for detecting novel objects and for exploring large ones with the fish's side using the back and forth propulsion system based on the smooth beat of the anal fin or body-bending around large objects. In this case, proprioceptive evaluation of body bending around an object may be clue for object shape. Dynamical analysis of sensory and motor behavior in this species is lacking and it must be performed to unveil the functional relationships between sensing and motor control and their role in perception [40].

### Local effects

The presence of edges causing local increases in current density will increase the importance of localized small dipoles. We refer to the images of these dipoles as “local” effects of the object. The larger the number of surface sinuosity, the larger number of smaller and differently oriented equivalent dipoles would appear. This explains the peaks observed in the images of the nail and objects with textured surface oriented with the edges towards the fish's body surface.

Local effects can only be sensed if their generating sources are close enough to the skin and can be correctly sampled if there is enough spatial resolution in the receptor mosaic. Because of their small norm and diverse orientation the relative weight of such dipoles in the far field is reduced. Objects cause multiple local effects when their surface is almost in contact with the skin. These features can only be sampled using a high resolution sensory mosaic. Then, surface electric features of an object are encoded by local effects on the fovea. Focusing on small objects near the mouth and exploring their surface and borders using small movements may be used for fine recognition of objects and preys.

A good foraging strategy might be exploring large objects in which local increases in conductance or capacitance inform the fish about the vitality of a prey attached to the surface [41]. Behavioral and physiological evidence of adaptive sensory responses taking place at the electrosensory lobe [42]–[44] suggest the presence of a background removal mechanism. Removing global effects may serve to enhance the contribution of the surface features of the object to the neural image.

### Conclusions

Fish's body and self-generated electric field geometries are embodied in the spatial profiles of active electric images. We describe two important contributions of the object stamp as potential clues for object recognition: “local” and “global” effects. These characteristics of active electrosensory images may generate object attributes similar to rugosity and hardness observed in touch. Active electroreception is a very short range sense for global effects and almost a contact sense for discriminating surface features. As in touch, fine exploration of surface texture and borders is enhanced by active changes in the points of view of a high resolution sensory mosaic and object hardness results from the coarse exploration based on the global action of the carrier. Also, as in touch, body bending around an object may provide clues on its shape. Because of these characteristics of active electrolocation, pulse gymnotiforms may recognize “adjacent objects with the active use of their own body” [45]. It could be speculated that, together with fish's proprioception and mechanical touch, electroreception may integrate a multiple-modal type of haptic sense.

## Materials and Methods

### Animals and general setup

Experiments were performed in 22 anesthetized fish (150 to 300 millimeters total length) following the guidelines of the CHEA (Comisión Honoraria de Experimentación Animal, ordinance 4332–99, Universidad de la República Oriental del Uruguay). Experiments were approved by the Animal Ethics Committee of the Instituto de Investigaciones Biológicas Clemente Estable (protocol number 001/03/2011). Fish were gathered at Laguna del Cisne (Maldonado, Uruguay) 1 to 4 months before the experiment, kept in individual aquaria under a natural light cycle and fed with insect larvae. Before the experiments, animals were injected with pentobarbital (0.5–1 milligrams, intramuscular) repeated on demand up to reach and maintain the EOD rate below 10 Hz at 20 degrees (C) and a slow but stable respiration. After the experiments animals were euthanized by pentobarbital (10 milligrams, intramuscular). At the anesthetic plane where the EOD rate was fixed unresponsive to visual, vibratory, electric or nociceptive stimuli, and after confirming the lack of motor responses to nociceptive stimuli, we implanted a cotton thread placed along the midline between the muscular masses and above the spine using a long steel needle. This thread came out of the body just behind the occiput and at the limit between the caudal and the center-caudal quarter of the fish length. The ends of this thread were firmly attached to wood poles (10 millimeters diameter both with a reduction to 3 millimeters below water level for the rostral one) hanging from an iron bar placed outside the tank. The rostral pole was placed just above the occiput. This allowed us to maintain the fish's body straight, halfway between the bottom and the water level in a 33×48 centimeters tank filled up to 10 centimeters with water (100±10 micro Siemens per centimeter).

### Methods for measuring the LEOD

The electric image of an element is the change in the transcutaneous field caused by the presence of such element in a given scene.

**Figure 10:**
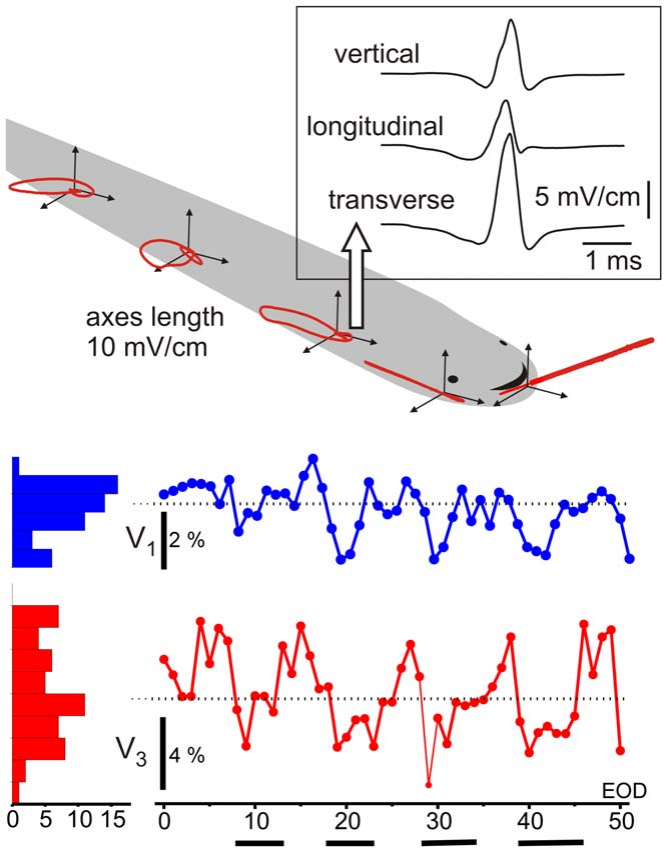
Methods. A) Local fields (LEOD) were recorded along three orthogonal directions. We measured the drop of voltage between a reference point placed adjacent to the skin and each three other points placed along orthogonal directions (longitudinal, transverse and vertical) and the fields components were estimated dividing the recorded drop of voltage by the inter-electrode distance. At the fovea the field vector moved along a line, while at the trunk the field showed wobbles due to the different origin of the EOD components. B) Changes in the amplitude of field components at the fovea due to the respiratory movements.

We used two different methods for evaluating the transcutaneous field: a direct measurement of transcutaneous local electric field (T-LEOD) between an internal and an external electrode and the measurement of the adjacent local field in water along the 3 orthogonal spatial dimensions of the field (3D-LEOD, Fig. 10).

In the case of T-LEOD, a 100 micrometers nichrome wire coated except on a knot tied at its center was percutaneously implanted. The wire entered through one side below the mandible and leaved symmetrically through the other side. After wire insertion we verified that the knot was at the center of the mandible, each side of the wire was bent up and above the head both were twisted together forming a ring around the head securing its position. Both ends of this reference electrode were connected together to one input of the differential amplifier. The exploring electrode (a 150 micrometers tungsten enamel coated electrode with a blunted bare tip) was placed at a distance of 1 millimeter from the skin at different points along a curve on the horizontal plane following the shape of the mandible. This electrode was connected to the other input of the differential amplifier.

In this species there is no electric organ (EO) at the head region and because of jaw curvature the distance between the reference and the exploring electrodes was similar all along the recording trajectory. We considered that the drop of voltage between exploring and reference electrode was a good estimator of the transcutaneous voltage at the fovea. Because of the complex geometry, and the multiplicity of sources causing the EOD along the caudal 90% of the fish's body, this method was not useful to evaluate the transcutaneous field at other regions.

In the case of the 3D-LEOD we measured the drop of voltage between a reference point placed adjacent to the skin and each three other points placed along orthogonal directions (longitudinal, transverse and vertical). Four tungsten enamel coated electrodes (150 micrometers diameter) assembled with their blunt tips placed at non coplanar points defining orthogonal lines (2 millimeters apart). This electrode assembly allowed us to measure the drop of voltage across the water in each direction and consequently to estimate the local electric field dividing the recorded drop of voltage by the interelectrode distance. Theoretical predictions indicate that when electrode assembly is placed against the skin, the recorded field is very similar to the transcutaneous field. Although this procedure allowed us to evaluate the electric image at the fovea and at the side of the fish body, it precluded placement of the exploring objects very close to the skin because of its large dimensions (the whole assembly occupies 2.2 millimeters of the space between the skin and the object). Since distances between skin and objects smaller than 2 millimeters are the most relevant for the image profile, T-LEODs was generally preferred for exploring LEOD profiles at the fovea.

### Experimental protocols and signal processing

Recordings of electric images were made by placing the objects at steady positions and moving the exploring electrode along a series of points on the skin with a step of 100 to 200 micrometers at the fovea and 2 millimeters on the side of the fish. Electrode tips were moved step-by-step along series of points on a previously defined plane using a computer controlled X-Y plotter (HP 7015A). This device allowed us a precise (less than 50 micrometers) control and recording of the active electrode position. Previously to the experiment, electrode trajectory was defined. On the side of the body equally spaced positions along a line following the skin were explored. At the fovea, the electrode trajectory was defined by hand-positioning the electrode at 50 sites along the curvature of the jaw. Positions of these points were recorded from the plotter reference output. We found the algebraic relationship between the plotter's input voltage and the position of the recording electrode, and applying this function we controlled the plotter movements through the sound card of a personal computer in house tuned for a DC-10000 kHz flat output.

Signals were digitized at least at 20 kHz per channel and amplified enough to have at least 12 bits resolution (AM systems-1800, 10-10000 Hz band pass). Data acquisition was made in epochs of 550–700 ms, starting 100 ms after the electrode movement ceased. Five to seven channels were recorded in each experiment: a) the head to tail EOD recorded between two electrodes placed on the main axis of the fish at opposite faces of the tank; b) the local field (either 3 channels of the 3D-LEOD or 1 channel of the T-LEOD), c) the X and Y positions of the electrode on the horizontal plane and d) a computer emitted trigger signal that started the acquisition after the electrode had reached each pre-programmed X-Y position. We made 3 to 5 runs of the electrode along the same trajectory with and without the object. For each of these runs, we obtained several (between 5 and 10) LEODs per recording position. LEOD waveforms were time averaged with reference to a fixed moment of the head to tail EOD. Thus, for each position we got 3 to 5 averaged LEOD waveforms.

Local field intensity profiles were estimated as the *rms* value of the LEODs (square root of the mean of the squared LEOD over 10 ms) in the presence and absence of an object. Median values were calculated and fitted by a smooth line calculated as a 3 or 5 points moving average of the median. Image profiles were obtained as the increments or modulation of the rms-value of local electric fields. Increment (Δ*rms*LEOD) and modulation are related according to the following expression.
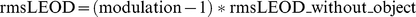



Finally, we showed the effect of the fish's body on the image profile in a qualitative experiment. We compared two field profiles. One was obtained along the jaw of a fish's cadaver; the other by placing the electrodes at the same points in the absence of the cadaver. Each profile consisted of the Δ*rms* caused by the presence of a metal cube facing the skin (or its equivalent surface) 3 millimeters away. We measured the local field with the 3D electrode array. The polarizing field was generated by a sine wave generator (1 kHz, 1 mV/cm) connected to the water through two large electrodes placed on the walls of the tank (facing the rostral and caudal poles of the fish respectively). The effect of the body was evaluated as the difference between the Δ*rms* profiles obtained with and without the presence of the object.
